# Hydrophilic, Red‐Emitting, and Thermally Activated Delayed Fluorescence Emitter for Time‐Resolved Luminescence Imaging by Mitochondrion‐Induced Aggregation in Living Cells

**DOI:** 10.1002/advs.201801729

**Published:** 2019-01-13

**Authors:** Fan Ni, Zece Zhu, Xiao Tong, Weixuan Zeng, Kebin An, Danqing Wei, Shaolong Gong, Qiang Zhao, Xiang Zhou, Chuluo Yang

**Affiliations:** ^1^ Department of Chemistry and Hubei Key Lab on Organic and Polymeric Optoelectronic Materials Wuhan University Wuhan 430072 China; ^2^ College of Materials Science and Engineering Shenzhen University Shenzhen 518060 China; ^3^ Wuhan National Laboratory for Optoelectronics Huazhong University of Science and Technology Wuhan 430074 China; ^4^ Key Laboratory for Organic Electronics & Information Displays and Institute of Advanced Materials Nanjing University of Posts and Telecommunications Nanjing 210023 China

**Keywords:** aggregation‐induced delayed fluorescence enhancement, mitochondria‐specific imaging, thermally activated delayed fluorescence, time‐resolved luminescence imaging

## Abstract

Thermally activated delayed fluorescence (TADF) materials have provided new strategies for time‐resolved luminescence imaging (TRLI); however, the development of hydrophilic TADF luminophores for specific imaging in cells remains a substantial challenge. In this study, a mitochondria‐induced aggregation strategy for TRLI is proposed with the design and utilization of the hydrophilic TADF luminophore ((10‐(1,3‐dioxo‐2‐phenyl‐2,3‐dihydro‐1H‐benzo[de]isoquinolin‐6‐yl)‐9,9‐dimethyl‐9,10‐dihydroacridin‐2‐yl)methyl)triphenylphosphonium bromide **(NID‐TPP)**. Using a nonconjugated linker to introduce a triphenylphosphonium (TPP^+^) group into the 6‐(9,9‐dimethylacridin‐10(9*H*)‐yl)‐2‐phenyl‐1H‐benzo[*de*]isoquinoline‐1,3(2*H*)‐dione **(NID)** TADF luminophore preserves the TADF emission of **NID‐TPP**. **NID‐TPP** shows clear aggregation‐induced delayed fluorescence enhancement behavior, which provides a practical strategy for long‐lived delayed fluorescence emission in an oxygen‐containing environment. Finally, the designed mitochondrion‐targeting TPP^+^ group in **NID‐TPP** induces the adequate accumulation of **NID‐TPP** and results in the first reported TADF‐based time‐resolved luminescence imaging and two‐photon imaging of mitochondria in living cells.

## 1 Introduction

Nonspecific and nonnegligible fluorescence signals derived from diverse endogenous luminophores in cells are obstacles for accurate and efficient cell imaging.^1^ Time‐resolved luminescence imaging (TRLI), a distinctive fluorescence imaging strategy for the detection of events occurring for long periods of time,^2^ can practically increase the signal‐to‐noise ratio by substantially eliminating background signals from scattering and autofluorescence.^3^ Typical TRLI modalities include phosphorescent transition metal (e.g., Ru(II),^4^ Ir(III),^5^ Pt(II),^6^ and Ln(III)^7^) complexes, in which long‐lived luminescence is mainly based on metal‐to‐ligand charge transfer or sensitized excited states of lanthanide ions. Alternatively, developing nonmetallic organic luminophores with long‐lived luminescence is also in great demand. Among the multifarious candidates, small‐molecule organic luminophores have constituted the majority of fluorescent emitters used in biological imaging because of their established chemistry, scalability, and diverse physical, chemical, and biological properties. However, due to the fast relaxation of the *S*_1_ state to the *S*_0_ state, most nonmetallic organic luminophores only exhibit short‐lived fluorescence (less than 10 ns). Although the phosphorescence (*T*_1_ state to *S*_0_ state) of these materials typically occurs for several milliseconds to even seconds, it is usually observed in solid states or at very low temperatures,^8^ which are usually not compatible with physiological conditions.^9^ Additionally, long‐lived fluorescence emissions observed in excimers and exciplexes have also been used for designing TRLI probes.^10^

Recently, thermally activated delayed fluorescence (TADF) emitters have rapidly emerged as some of the most attractive luminescent materials.^11^ They exhibit delayed fluorescence with lifetimes ranging from nanoseconds to milliseconds, which are comparable with those of transition metal complexes. The long‐lived fluorescence resulting from the reversed intersystem crossing (RISC) from the triplet excited state (*T*_1_) to the singlet excited state (S_1_) can sensibly provide a new strategy for TRLI. Although substantial progress has been made in organic light‐emitting diode devices by using TADF materials,^12^ few of the TADF emitters have been utilized for biological imaging.^13^

The use of TADF luminophores for TRLI in cells is limited because of their sensitive *T*_1_ state, which is easily quenched by surrounding oxygen.[[qv: 12a,d]] Based on these preconditions, reducing the triplet quenching by oxygen has become a crucial issue to be resolved for TRLI in cells (Table S1, Supporting Information). Efficient strategies have been developed to realize TADF‐based TRLI by introducing additional assistance or pre‐embedding.[[qv: 13a,b,d,e–g]] And our previous design of the in situ hydrophobic aggregation of TADF luminophores has also been proved to be an alternative method.[[qv: 13h]] The exploitation of TADF‐based probes has been exemplified in the fabrication of aggregates of hydrophobic luminophores,[[qv: 13d,f–h]] where the contact between TADF molecules with oxygen can be reduced during the aggregation process and the triplet–triplet energy transfer from the TADF luminophore aggregates to surrounding oxygen has been restrained by aggregation‐induced delayed fluorescence behavior. As a result, the desired delayed fluorescence emission has been obtained in living cells for TRLI. Despite the elegant design of these TADF luminophores, their intrinsic hydrophobicity is still an obstacle for obtaining access into cells. The need to develop hydrophilic luminophores with TADF features is important because hydrophilic luminophores can not only be more compatible in cells but also theoretically afford opportunities for multifunctional sensing and detection, such as the targeting of organelles,^14^ DNA,^15^ proteins,[[qv: 2c,16]] and small molecules of biological interest^17^ and sensing cellular macro/microenvironments.^18^ However, hydrophilic luminophores are much easier to disperse as a single molecular state so that the long‐lived TADF emission cannot be obtained through the approach of the self‐aggregation behavior, as the hydrophobic TADF luminophores have demonstrated. Until now, the use of corresponding luminophores for simultaneous TRLI and functional sensing or detection remains difficult.

**Scheme 1:**
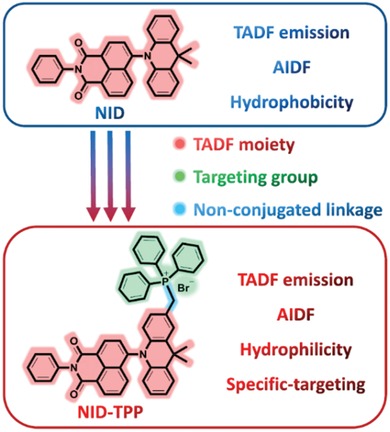
The design of the probe **NID‐TPP** for TRLI.

In this study, a new design of mitochondria‐targeted and induced aggregation of hydrophilic TADF luminophores for TRLI was proposed. The hydrophilic TADF luminophore ((10‐(1,3‐dioxo‐2‐phenyl‐2,3‐dihydro‐1H‐benzo[de]isoquinolin‐6‐yl)‐9,9‐dimethyl‐9,10‐dihydroacridin‐2‐yl)methyl)triphenylphosphonium bromide **(NID‐TPP)** was designed and synthesized through a simple four‐step synthesis (**Scheme** **1** and Section S1, Supporting Information). The core TADF‐featured luminophore 6‐(9,9‐dimethylacridin‐10(9*H*)‐yl)‐2‐phenyl‐1H‐benzo[*de*]isoquinoline‐1,3(2*H*)‐dione **(NID)** was first constructed using 9,9‐dimethyl‐9,10‐dihydroacridine (DMAC) and 1,8‐naphthalimide as the electron donor and acceptor, respectively.^19^ A triphenylphosphonium (TPP^+^) group was incorporated to modify the **NID** luminophore through a nonconjugated methylene linking group to change the hydrophobic TADF luminophore into a hydrophilic TADF luminophore (Scheme 1). **NID‐TPP** in a single molecule state demonstrated very weak emission and no TADF features even in a degassed atmosphere but showed obvious aggregation‐induced delayed fluorescence enhancement (AIDFE) behavior in an aerated atmosphere, which led to oxygen‐containing TADF emission and TRLI in living cells. It is expected that **NID‐TPP** decorated with a TPP^+^ group not only provides a resolution for water solubility but also offers the possibility for the targeting of and accumulation in mitochondria to achieve the desired TADF emission and TRLI in living cells.^20^

## 2 Results and Discussion

### 2.1 The TADF and AIDFE Features of NID and NID‐TPP

First, to estimate the involvement of the TADF emission in **NID‐TPP**, the ground‐state geometry of the core moiety **NID** was simulated by density functional theory with the B3LYP functional (Figure S1, Supporting Information). The DMAC unit was markedly distorted from the naphthalene ring by bulk steric hindrance, leading to a small overlap between the highest occupied molecular orbital and the lowest unoccupied molecular orbital and a small singlet‐triplet splitting energy (Δ*E*_ST_) value as low as 0.23 eV. These calculations agreed well with the strategy for designing TADF molecules with an effective RISC.^11^

**Figure 1:**
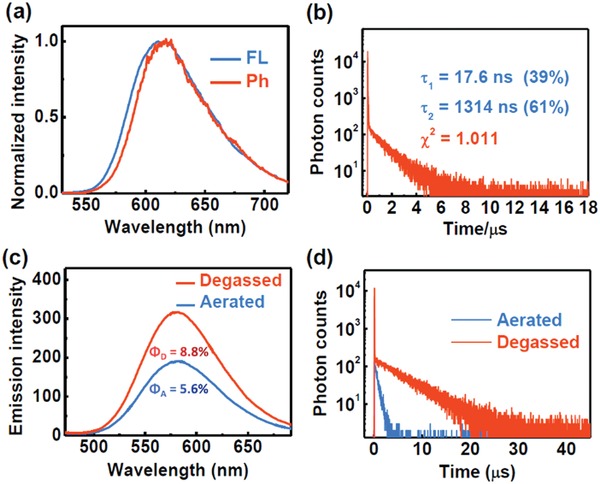
a) The fluorescence and phosphorescence emission spectra of **NID** in solid state at 77 K. λ_ex_ = 365 nm. b) The transient photoluminescence decay spectra of **NID** in solid state at room temperature. λ_ex_ = 377 nm, λ_em_ = 610 nm. c) The steady‐state emission spectra of **NID** in toluene solution at room temperature under aerated and degassed atmosphere. λ_ex_ = 365 nm. d) The transient photoluminescence decay spectra of **NID** in toluene solution at room temperature under aerated and degassed atmosphere. [**NID**] = 50 × 10^−6^ m. λ_ex_ = 377 nm, λ_em_ = 581 nm.

By measuring the fluorescence and phosphorescence spectra of **NID** in the solid state at 77 K, the singlet and triplet state energies were estimated to be 2.19 and 2.16 eV, respectively (**Figure** **1**a). The Δ*E*_ST_ of 0.03 eV was small enough for **NID** to realize the RISC process.

The transient photoluminescence decay spectra of **NID** in the solid state (Figure 1b) and in degassed toluene solution (Figure 1d) both showed biexponential fluorescence decay with a short lifetime in the nanosecond range and a long lifetime in the microsecond range. The long‐lived fluorescent component in toluene solution decayed slowly after degassing with argon (τ_2_ = 5.58 µs). The discrepancies coincided with the change in fluorescence intensity with and without degassing (Figure 1c). These results confirmed the TADF features of **NID**.

**Figure 2:**
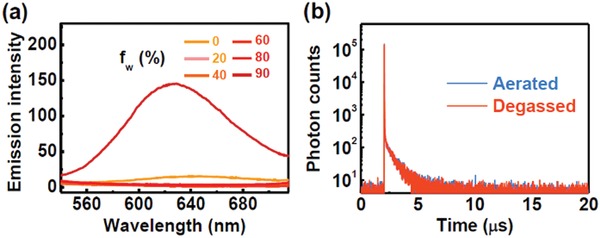
a) Steady‐state emission spectra of **NID** in THF and water mixtures at different *f*_w_ under aerated atmosphere. λ_ex_ = 365 nm. b) The transient photoluminescence decay spectra of **NID** in THF/water (*f*_w_ = 90%) mixtures at room temperature under aerated and degassed atmosphere. [**NID**] = 50 × 10^−6^ m. λ_ex_ = 377 nm, λ_em_ = 627 nm.

The steady‐state emission spectra of **NID** in tetrahydrofuran (THF)/water mixtures with different volume ratios were investigated under an aerated atmosphere. When the volume ratio of water (*f*_w_) increased to 90%, the solution became turbid and the fluorescence intensity increased significantly (**Figure** **2**a). A typical aggregation‐induced emission enhancement (AIEE) phenomenon was observed,^21^ in which the nonradiative pathway was inhibited because of the restriction of intramolecular rotations in the aggregated state of **NID**. In addition, the transient photoluminescence decay spectra of **NID** in THF/water (*f*_w_ = 90%) mixtures revealed a long‐lived lifetime in the microsecond range. The lifetimes under aerated and degassed atmospheres were estimated to be 0.82 and 0.83 µs, respectively. The near overlap between the two decay curves and the approximately equivalent lifetimes indicated that the TADF emissions of the aggregates were not sensitive to oxygen (Figure 2b), which demonstrated the ability for aggregates of TADF luminophores to conduct TRLI.

Based on these results, **NID**, which demonstrated obvious aggregation‐enhanced TADF features and orange‐red emission, is an ideal candidate for designing probes for time‐resolved luminescence imaging. To improve the hydrophilicity of **NID**, a TPP^+^ group was incorporated into the **NID** luminophore to obtain the modified **NID‐TPP** probe (Figure S2a, Supporting Information). A nonconjugated connection between the **NID** and TPP^+^ moieties was used to not influence the luminescence properties, especially for the TADF emission. Moreover, the cationic TPP^+^ group can promote the intracellular accumulation of the **NID‐TPP** probe in the mitochondrial matrix,[[qv: 14b]] which may lead to an increase in the concentration of **NID‐TPP** in mitochondria, thus enabling efficient TADF emission and TRLI in living cells.

**Figure 3:**
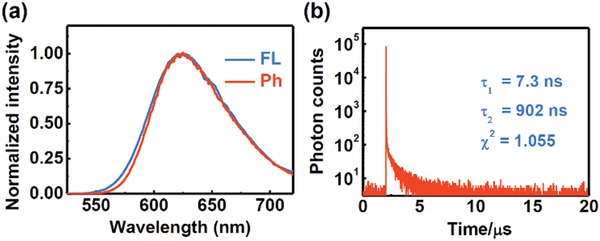
a) The fluorescence and phosphorescence emission spectra of **NID‐TPP** in solid state at 77 K. λ_ex_ = 365 nm. b) The transient photoluminescence decay spectra of **NID‐TPP** in solid state at room temperature. λ_ex_ = 377 nm, λ_em_ = 625 nm.

The emission spectrum of **NID‐TPP** in the solid state at 77 K was similar to that of **NID** (**Figure** **3**a), which suggested that the nonconjugated introduction of the TPP^+^ group did not affect the excited energy of **NID‐TPP**. The single state and triplet state energies were estimated to be 2.18 and 2.15 eV, respectively (Figure 3a), with a sufficiently small Δ*E*_ST_ value of 0.03 eV.

**Figure 4:**
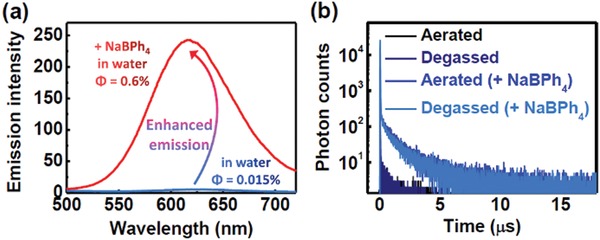
a) The steady‐state emission spectra of the **NID‐TPP** aqueous solution in the absence and presence of NaBPh_4_ at room temperature under an aerated atmosphere. λ_ex_ = 365 nm. b) The transient photoluminescence decay spectra of the **NID‐TPP** aqueous solution in the absence and presence of NaBPh_4_ at room temperature under aerated and degassed atmospheres. [**NID‐TPP**] = [NaBPh_4_] = 50 × 10^−6^ m. λ_ex_ = 377 nm, λ_em_ = 618 nm.

The transient photoluminescence decay spectrum of **NID‐TPP** in the solid state also revealed a short‐lived lifetime (τ = 7.3 ns, Figure 3b) and a long‐lived lifetime (τ = 902 ns, Figure 3b). The fluorescence intensity of **NID‐TPP** in aqueous solution was too weak (**Figure** **4**a), and its transient photoluminescence decay spectra either with or without degassing both showed no TADF emission. The photoluminescence quantum yield of the **NID‐TPP** aqueous solution was 0.015% compared with that of quinine sulfate in 0.1 m H_2_SO_4_ (Φ = 54%).^22^ Due to the mutual electrostatic repulsion, we speculated that **NID‐TPP** in water is in a single molecule state, and its excited energy can be consumed through intramolecular rotation, which leads to weak luminescence. To verify this hypothesis, the **NID‐TPP** solution was added to sodium tetraphenylborate and then became turbid. The luminescence quantum yield increased 40‐fold compared with its initial value (Φ = 0.6%, Figure 4a). Due to the strong electrostatic interactions between the TPP^+^ group and tetraphenylborate, **NID‐TPP** aggregated, which restrained the free rotation that improved the strong emission (Figure S2b,c, Supporting Information). The measured transient photoluminescence decay spectra of the resulting suspension revealed biexponential fluorescence decays with a short lifetime in the nanosecond range (8.9 ns, Figure S3, Supporting Information) and a long lifetime in the microsecond range (1.2 µs, Figure S3, Supporting Information). Moreover, the long‐lived fluorescence was not sensitive to oxygen (Figure 4b). Notably, **NID‐TPP** only showed weak emission with no TADF features in the aggregation‐free state but showed noticeably stronger emission and TADF features in the aggregated state.

**Figure 5:**
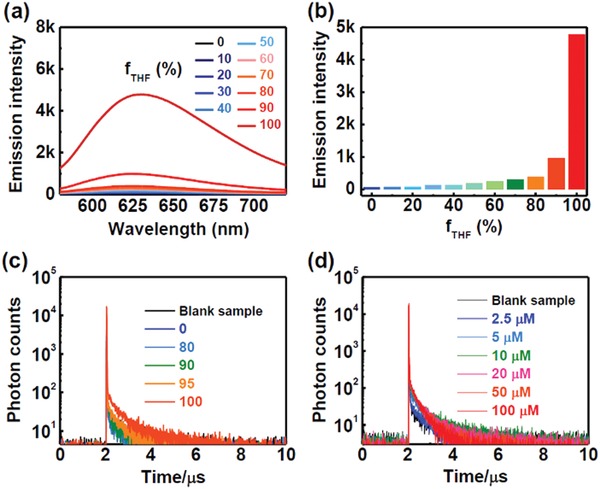
a) The steady‐state emission spectra of 10 × 10^−6^ m **NID‐TPP** in DMSO/THF mixtures at different *f*_THF_ under an aerated atmosphere. λ_ex_ = 250 nm. b) The emission intensity at a wavelength of 625 nm with different *f*_w_. λ_ex_ = 250 nm. c) The transient photoluminescence decay spectra of **NID‐TPP** in DMSO/THF mixtures with different *f*_THF_ under an aerated atmosphere. λ_ex_ = 250 nm. d) The transient photoluminescence decay spectra of different concentrations of **NID‐TPP** in DMSO/THF mixtures (*f*_w_ = 90%) under an aerated atmosphere. λ_ex_ = 377 nm, λ_em_ = 625 nm.

To further gain insight into the relationship between the emission features and the aggregation degree, the steady‐state emission spectra and transient photoluminescence decay spectra of **NID‐TPP** in dimethyl sulfoxide (DMSO)/THF mixtures with different volume ratios were investigated under an aerated atmosphere. The steady‐state emission demonstrated typical AIEE behavior with the continuously increasing volume ratio of THF (*f*_THF_) (**Figure** **5**a,b). With the increase in *f*_THF_, the ratios of the delayed components increased, demonstrating aggregation‐induced TADF emission enhancement behavior (Figure 5c). Then, the transient photoluminescence decay spectra of **NID‐TPP** with different concentrations were also measured. When **NID‐TPP** with a low concentration of 2.5 × 10^−6^ m was used, no noticeable TADF emission was detected. With the gradual increase in the concentration of **NID‐TPP**, the ratio of the delayed components also showed an increase (Figure 5d). These results indicated that **NID‐TPP** demonstrated obvious AIDFE behavior, which offers a practical strategy for activating the TADF emission by the aggregation of **NID‐TPP** for TRLI in an oxygen‐containing environment.

### 2.2 Time‐Resolved Luminescence Imaging with NID‐TPP in HeLa Cells

Despite the reported results, the hydrophilic aggregation‐free state and the electrostatic repulsion in **NID‐TPP** still restrained the formation of the TADF aggregates for TRLI in cells. To overcome this issue, a mitochondria‐induced aggregation strategy was proposed. Due to the reduced and more negative inside membrane potential in plasma and mitochondria, the concentration of positively charged species, such as **NID‐TPP**, can gradually accumulate in cytoplasm and further into the mitochondrial matrix through an approach of passive transport. Moreover, the cationic compounds can be 100‐ to 1000‐fold or more concentrated in the mitochondrial matrix than in the extracellular envrionment.[[qv: 14b]] To prove this, the uptake efficiency of **NID‐TPP** in cellular environment was determined by flow cytometry assay (Figure S6, Supporting Information). All of the HeLa cells incubated with **NID‐TPP** exhibited significantly higher fluorescence intensity than the control cells, which clearly demonstrated the efficient cellular uptake of **NID‐TPP**.

**Figure 6:**
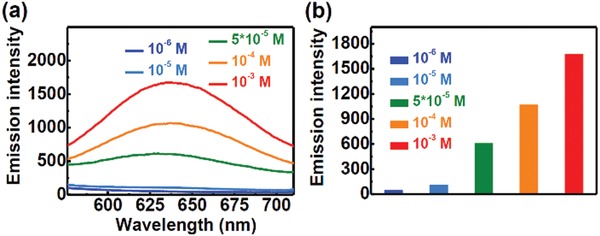
a) The steady‐state emission spectra of different concentrations of **NID‐TPP** in water under an aerated atmosphere. λ_ex_ = 377 nm. b) The emission intensity with different concentrations of **NID‐TPP** in water. λ_ex_ = 377 nm, λ_em_ = 637 nm.

Then, the steady and transient photoluminescence spectra of the aqueous solution of **NID‐TPP** with different concentrations were measured under an aerated atmosphere (**Figure** **6**). With the increase in the concentration, the nonradiative transition of free rotation was restricted, and the energy transfer from the triplet state of **NID‐TPP** to triplet oxygen was restrained, which resulted in the gradual enhancement of the steady emission (Figure 6), and the long‐lived delayed fluorescence was gradually activated in an oxygen‐containing environment (Figure S7, Supporting Information). These positive results indicated that the high concentration‐induced aggregation can block the triplet energy loss pathway in **NID‐TPP** aggregates to allow mitochondria‐induced aggregation of **NID‐TPP** to also contribute to the TADF emission in living cells.

**Figure 7:**
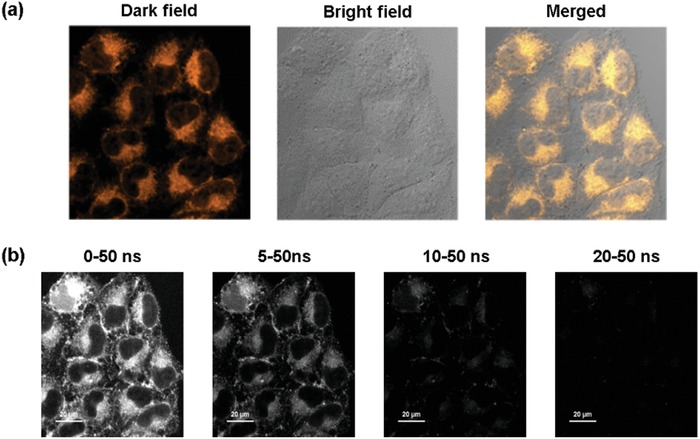
a) Steady‐state and b) time‐gated luminescence images of HeLa cells incubated with 10 × 10^−6^ m **NID‐TPP** for 2 h. λ_ex_ = 405 nm, λ_em_ = 570–670 nm.

To further substantiate the roles of the likely mitochondria‐induced aggregation in cells, the luminophore **NID‐TPP** was utilized for time‐resolved luminescence imaging. HeLa cells were rinsed three times with clean phosphate buffer saline (PBS) to confirm that the probe permeated the cell membrane. Luminescence signals were observed in the cytoplasm rather than in the nuclear region (**Figure** **7**). Time‐resolved luminescence imaging showed that evident luminescence could also be observed after exerting a delay time greater than 10 ns (Figure 7b).

**Figure 8:**
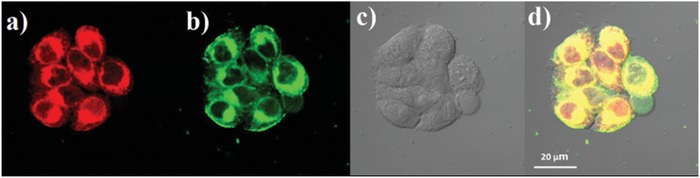
Costaining of **NID‐TPP** (10 × 10^−6^ m) with MitoTracker Green (1 × 10^−6^ m) in HeLa cells. a) A luminescent image of **NID‐TPP**. λ_ex_ = 405 nm, λ_em_ = 570–670 nm. b) A luminescent image of MitoTracker Green. λ_ex_ = 488 nm, λ_em_ = 500–550 nm. c) A bright‐field image. d) A merged image of images panels (a–c).

The cytotoxicity of **NID**, **NID‐TPP**, and the commercially available mitochondria‐specific staining probe MitoTracker Green was measured by 3‐(4,5‐dimethyl‐2‐thiazolyl)‐2,5‐diphenyl‐2‐H‐tetrazolium bromide (MTT) cell proliferation test, which demonstrated the better biocompatibility of **NID‐TPP** than MitoTracker Green (Figure S5, Supporting Information). To confirm the specific staining in mitochondria, a colocalization staining experiment was performed with the staining probe MitoTracker Green[[qv: 19c]] (**Figure** **8**). The emission image constructed from the orange to red wavelength window (570–670 nm) overlapped well with that from the green wavelength window (500–550 nm). A colocalization coefficient (Pearson's correlation) of 0.91 demonstrated that the fluorescence of **NID‐TPP** was mainly distributed in mitochondria. Furthermore, due to the AIDFE behavior of **NID‐TPP**, staining with **NID‐TPP** showed no signal in the extracellular environment and weak signals in the cytoplasm but strong signals in mitochondria compared with the imaging results using MitoTracker Green (Figure 8d). These observations further confirmed the AIDFE feature of **NID‐TPP** and the advantages of **NID‐TPP**, suggesting an even more accurate probe for mitochondrial imaging.

**Scheme 2:**
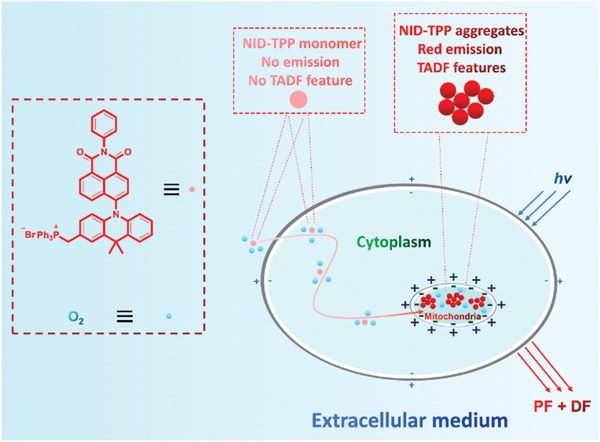
The proposed mechanism of **NID‐TPP** for TRLI of mitochondria in HeLa cells.

In order to exclude the possibility of the nonspecial binding in cells, the emission spectra of **NID‐TPP** in the aqueous solutions with different anions in very high concentration were also investigated, where no emission signal can be detected (Figure S2b,c, Supporting Information). It can be concluded that the anions existed in cells cannot cause the aggregation and emission of **NID‐TPP**. The emission spectra of **NID‐TPP** in the aqueous solutions with bovine serum albumin or fetal bovine serum in very high concentration were also measured, while no obvious red emission can be detected. This suggests that the nonspecific hydrophobic interaction between biomatrix and **NID‐TPP** cannot provide the channel for the effective emission (Figure S8, Supporting Information). Based on these results, we believe that the design of the mitochondria‐induced aggregation to activate TADF emission for TRLI is reasonable (**Scheme** **2**).

Two‐photon luminescence imaging featured with weak autofluorescence and self‐absorption, deep *z*‐axis depth‐of‐field, and reduced photodamage to cells can be an effective approach for bioimaging. Among the candidates for effective two‐photon luminescence imaging, 1,8‐naphthalimide‐based emitters have shown great advantages in efficient two‐photon properties.^23^ Therefore, the potential application of the two‐photon microscopic technique with **NID‐TPP** in HeLa cells was also explored. As to simulate the aggregation state of **NID‐TPP** in mitochondria, the aqueous solution of 50 × 10^−6^ m **NID‐TPP** after the addition of 50 × 10^−6^ m NaBPh_4_ was used. Under the two‐photon excitation mode, the formed aggregates in water demonstrated the absorption maxima at 720 and 820 nm in water (Figure S9a, Supporting Information). The maximal two‐photon action cross‐sectional values (*Φδ*_max_s) were determined to be 729 GM at 720 nm and 625 GM at 820 nm. Excited with 820 nm two‐photon pulse laser, the aggregates presented almost the same emission spectrum with that under one‐photon mode (Figure S9b, Supporting Information).

**Figure 9:**
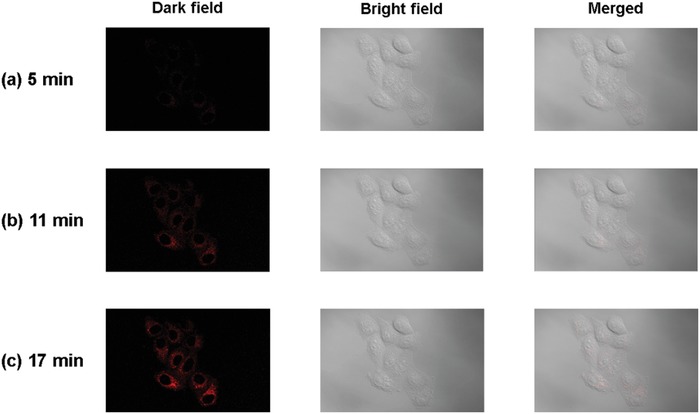
Two‐photon luminescent images of HeLa cells incubated with 10 × 10^−6^ m **NID‐TPP** for a) 5 min, b) 11 min, and c) 17 min. λ_ex_ = 810 nm, λ_em_ = 540–660 nm.

With the above positive results in hand, the two‐photon luminescence imaging of HeLa cells after labeled with **NID‐TPP** was explored. The HeLa cells were incubated with 10 × 10^−6^ m **NID‐TPP** at 37 °C and then were directly subjected to two‐photon fluorescence imaging without washing with phosphate buffer. The recorded images were presented in **Figure** **9**. Due to the AIDFE behavior of **NID‐TPP**, no fluorescence signal can be detected in extracellular medium. With lengthening the incubation time, enhanced fluorescence signals can be observed in HeLa cells, demonstrating that **NID‐TPP** is also a suitable candidate for two‐photon luminescence imaging.

## 3 Conclusion

In conclusion, we developed a hydrophilic TADF emitter consisting of **NID‐TPP** with red emission and AIDFE behavior, in which the likely triplet energy‐loss in vitro was inhibited by its aggregation. Considering the established AIDFE feature and as to inhibit the dispersion of hydrophilic **NID‐TPP** in cells, a strategy for mitochondria‐induced aggregation of **NID‐TPP** was demonstrated. The TRLI process was conducted in HeLa cells, and the red TADF emission of **NID‐TPP** was specifically detected in mitochondria with decreased background signals by imaging within the specified time domain. This study revealed that well‐designed TADF‐based hydrophilic luminophores and organelle‐induced aggregation are very promising for time‐resolved luminescence imaging and two‐photon luminescence imaging in oxygenated environments and organisms.

## Conflict of Interest

The authors declare no conflict of interest.

## Supporting information

Supplementary
