# Supplementary material for: Hydrophilic, Red‐Emitting, and Thermally Activated Delayed Fluorescence Emitter for Time‐Resolved Luminescence Imaging by Mitochondrion‐Induced Aggregation in Living Cells
Source: Adv Sci (Weinh). 2019 Jan 13;6(5):1801729. doi: 10.1002/advs.201801729 (PMC6402405; doi:10.1002/advs.201801729)
Supplement: Supplementary file 1 — Supplementary [file ADVS-6-1801729-s001.pdf]

Copyright WILEY-VCH Verlag GmbH & Co. KGaA, 69469 Weinheim, Germany, 2019.

ADVANCED SCIENCE
Open Access

Supporting Information

for *Adv. Sci.,* DOI: 10.1002/advs.201801729

Hydrophilic, Red-Emitting, and Thermally Activated Delayed Fluorescence Emitter for Time-Resolved Luminescence Imaging by Mitochondrion-Induced Aggregation in Living Cells

Fan Ni, Zece Zhu, Xiao Tong, Weixuan Zeng, Kebin An, Danqing Wei, Shaolong Gong, Qiang Zhao, Xiang Zhou,* and Chuluo Yang**

Copyright WILEY-VCH Verlag GmbH & Co. KGaA, 69469 Weinheim, Germany, 2019.

Supporting Information

Hydrophilic, red-emitting, and thermally activated delayed fluorescence emitter for time-resolved luminescence imaging by mitochondrion-induced aggregation in living cells

Fan Ni,[‡] Zece Zhu,[‡] Xiao Tong, Weixuan Zeng, Kebin An, Danqing Wei, Shaolong Gong, Qiang Zhao,[*] Xiang Zhou[*] and Chuluo Yang[*]

Table of contents

Experimental Procedures **S3-S6**

1. General information **S3**

2. Synthesis of NID and NID-TPP **S4-S7**

2.1. Synthesis of **1** S4

2.2. Synthesis of **NID** S5

2.3 Synthesis of **2**

S6

2.4 Synthesis of **NID-TPP**

S7

3. Determination of photoluminescence quantum yield

S8

4. Preparation of the nanoparticles **S8**

5. Cell imaging **S9**

6. Cytotoxicity test

S10

7. Flow cytometry analysis **S11**

The supplementary figures, table and spectra

S12-S20

References **S21**

Experimental Procedures

1. General information

^{1}H NMR and ^{13}C NMR spectra were collected on a MERCURY-VX300 or Bruker AVANCE III spectrometer. HRMS were obtained on Thermo Scientific LTQ Orbitrap XL. UV-vis absorption spectra were recorded on a Shimadzu UV-2700 recording spectrophotometer. Fluorescence and phosphorescence emission spectra at room temperature or 77K were measured on a Hitachi F-4600 spectrophotometer. The transient photoluminescence decay spectra were measured on an Edinburgh FLS920 spectrophotometer equipped with a picosecond pulsed diode laser (EPL-375, wavelength: 377nm; pulse width: 79.6 ps). The particle sizes were measured with Nano-ZS ZEN3600 Instruments. The used solvents of toluene and THF were dried through standard procedure. The other solvents and reagents used were purchased form commercial sources and used as received without further purication. E_S and E_T were calculated from the onset of the fluorescence spectra and the phosphorescence spectra, respectively.

2. The Synthesis of NID and NID-TPP

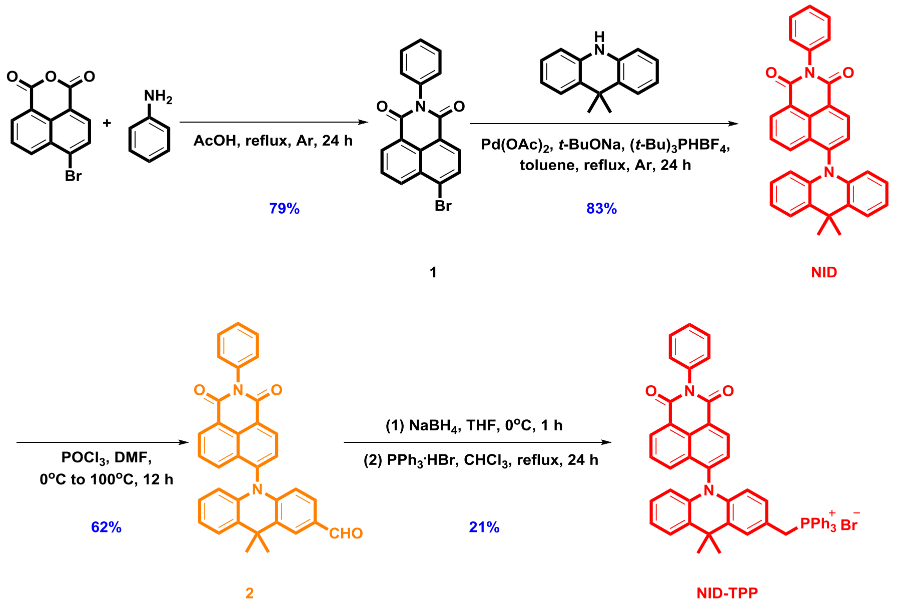

Scheme S1. The synthetic route for **NID** and **NID-TPP**

2.1 Synthesis of 6-bromo-2-phenyl-1H-benzo[de]isoquinoline-1,3(2H)-dione (1)

Compound **1** was synthesized through known method.[1] To a 100 mL round-bottom flask containing 4-bromo-1,8-naphthalic anhydride (5.54 g, 20 mmol) in 50 mL of acetic acid, aniline (2.79 g, 30 mmol) was added. The mixture was refluxed for 24 hours under the condition of argon. After the reaction was complete, the mixture was poured into ice-water. The resulting precipitate was filtered, washed by water and then purified by recrystallization from $CHCl_3$. A light-yellow powder **1** was obtained in 79% yield. ^{1}H NMR (300 MHz, $CDCl_3$) δ (ppm): 7.32 (d, J = 7.2 Hz, 2H), 7.47-7.58 (m, 3H), 7.87 (t, J = 6.6 Hz, 1H), 8.05 (d, J = 6.3 Hz, 1H), 8.43 (d, J = 7.2 Hz, 1H), 8.61 (d, J =8.1 Hz, 1H), 8.68 (d, J = 6.0 Hz, 1H).

2.2. Synthesis of 6-(9,9-dimethylacridin-10(9H)-yl)-2-phenyl-1H-benzo[de]-isoquinoline-1,3 (2H)-dione (NID)

NID was synthesized through known method.[2] 6-bromo-2-phenyl-1H-benzo[de]isoquinoline-1,3(2H)-dione (4.23 g, 12.0 mmol) (**1**), 9,9-dimethyl-9,10- dihydroacridine (2.76 g, 13.2 mmol), palladium(II) acetate (54 mg, 0.24 mmol), tri-tert-butylphosphine tetrafluoroborate (209 mg, 0.72 mmol) and sodium tert-butoxide (1.27 g, 15 mmol) were added to dry toluene (30 mL). The mixture was stirred at reflux for 24 hours then the cooled mixture was concentrated in vacuum. The crude product was purified by column chromatography with CH_2Cl_2 as eluent, finally the red powder **NID** was obtained with a yield of 83%. ^{1}H NMR (300 MHz, $CDCl_3$) δ (ppm):1.78 (s, 3H), 1.86 (s, 3H), 5.99 (d, J = 8.1 Hz, 2H), 6.88 (t, J = 7.5 Hz, 2H), 6.97 (t, J = 7.5 Hz, 2H), 7.37 (d, J = 7.2 Hz, 2H), 7.52-7.62 (m, 5H), 7.68 (t, J = 7.8 Hz, 1H), 7.84 (d, J = 7.8 Hz, 1H), 8.10 (d, J = 8.1 Hz, 1H), 8.69 (d, J = 6.6 Hz, 1H), 8.87 (d, J = 7.8 Hz, 1H). ^{13}C NMR (75 MHz, $CDCl_3$) δ (ppm): 32.1, 32.7, 36.0, 113.9, 121.3, 123.0, 123.8, 125.9, 126.7, 128.1, 128.5, 128.8, 129.4, 129.9, 130.4, 130.6, 130.8, 132.3, 132.9, 135.2, 140.0, 144.4, 163.7, 164.0. EI-MS: m/z $[M]^+$: 480.2, $[M-CH_3]^+$ 465.3.

2.3. Synthesis of 10-(1,3-dioxo-2-phenyl-2,3-dihydro-1H-benzo[de]isoquinolin-6-yl)-9,9-dimethyl-9,10-dihydroacridine-2-carbaldehyde (2)

NID (3.84 g, 8 mmol) in 35 mL dry DMF was kept at 0°C in argon atmosphere, $POCl_3$ (2.45 g, 16 mmol) was then added slowly. The mixture was stirred at 25°C for 1 hour and then at reflux for another 10 hours. After cooling down to room temperature, the mixture was slowly poured into 100 mL ice water, neutralized with 5% NaOH aqueous solution, and extracted with CH_2Cl_2 (100 mL × 3). The collected organic phase was dried over anhydrous Na_2SO_4, filtered and concentrated under reduced pressure. The crude product was purified by column chromatography with CH_2Cl_2 as eluent, the orange powder **2** was obtained with a yield of 62% (2.52 g, 4.96 mmol). ^{1}H NMR (400 MHz, $CDCl_3$) δ (ppm): 1.81 (s, 3H), 1.92 (s, 3H), 6.03 (d, J = 8.4 Hz, 1H), 6.09 (d, J = 8.4 Hz, 1H), 6.93 (t, J = 8.4 Hz, 1H), 7.06 (t, J = 8.0 Hz, 1H), 7.36-7.40 (m, 3H), 7.53 (t, J = 7.6 Hz, 1H), 7.58-7.63 (m, 3H), 7.72 (t, J = 8.0 Hz, 1H), 7.86 (d, J = 8.0 Hz, 1H), 8.02 (d, J = 8.4 Hz, 1H), 8.10 (s, 1H), 8.72 (d, J = 7.2 Hz, 1H), 8.90 (d, J = 7.6 Hz, 1H), 9.84 (s, 1H). ^{13}C NMR (100 MHz, $CDCl_3$) δ (ppm): 32.2, 33.1, 36.1, 114.0, 114.7, 122.8, 123.6, 123.9, 126.1, 127.1, 128.0, 128.5, 128.9, 129.5, 129.7, 129.8, 129.98, 130.00, 130.1, 130.3, 130.4, 130.5, 132.5, 132.8, 135.0, 138.8, 143.0, 144.8, 163.5, 163.8, 190.6. EI-MS: m/z $[M]^+$: 508.3, $[M-CH_3]^+$ 493.2.

2.4. Synthesis of ((10-(1,3-dioxo-2-phenyl-2,3-dihydro-1H-benzo[de]isoquinolin-6-yl)-9,9-dimethyl-9,10-dihydroacridin-2-yl)methyl)triphenylphosphonium bromi-de (NID-TPP)

2 (1.53 g, 3 mmol) in 20 mL dry THF was cooled down to 0°C and $NaBH_4$ (3.3 mmol, 125 mg) was then added slowly. The mixture was stirred at 0°C for another 1 hour till the disappearance of **2**. The mixture was slowly poured into 100 mL water and extracted with CH_2Cl_2 (100 mL × 3). The collected organic phase was dried over anhydrous Na_2SO_4, filtered and concentrated under reduced pressure. The crude product with triphenylphosphine hydrobromide (686 mg, 2 mmol) in 30 mL $CHCl_3$ was stirred at reflux for 24 hours and then concentrated under reduced pressure. The crude product was purified by column chromatography with $CHCl_3$/MeOH (v/v = 50/1) as eluent, the red powder **NID-TPP** was obtained with a yield of 21% (527 mg, 0.63 mmol) ^1H NMR (400 MHz, DMSO-d_6, ppm): 1.43 (s, 3H), 1.49 (s, 3H), 4.99-5.12 (m, 2H), 5.76 (d, J = 8.4 Hz, 1H), 5.92 (d, J = 7.6 Hz, 1H), 6.52 (d, J = 8.0 Hz, 1H), 6.89-6.96 (m, 2H), 7.16 (s, 1H), 7.43 (d, J = 6.8 Hz, 1H), 7.50-7.66 (m, 11H), 7.74-7.75 (m, 6H), 7.88-7.92 (m, 4H), 7.99 (d, J = 7.6 Hz, 1H), 8.57-8.58 (m, 1H), 8.73 (d, J = 7.2 Hz, 1H). ^{13}C NMR (100 MHz, DMSO-d_6, ppm): 32.1, 33.0, 35.6, 113.8, 114.0, 117.6, 118.4, 119.5, 119.6, 121.6, 123.6, 124.2, 126.5, 127.1, 128.4, 128.8, 129.1, 129.2, 129.3, 129.7, 130.1, 130.3, 130.4, 131.0, 131.5, 132.4, 134.1, 134.2, 135.1, 136.0, 139.3, 139.5, 142.6, 163.3, 163.6. HRMS (ESI): m/z calcd for $C_{52}H_{40}N_2O_2P^+$ $[M-Br]^+$ 755.2827, found 755.2843.

3. Determination of photoluminescence quantum yield

The photoluminescence quantum yields of fluorescence were determined by comparison of the integrated area of the emission spectra of the samples with the reference of Quinine sulfate in 0.1 M H_2SO_4 (Φ = 54%).[3] The photoluminescence quantum yields were calculated with the following expression:

$$\mathbf{\Phi_x = \Phi_{st} (I_x/I_{st}) (A_{st}/A_x)}$$

$\mathbf{\Phi_{st}}$ is the reported photoluminescence quantum yield of the standard, I is the area under the emission spectra, **A** is the absorbance at the excitation wavelength 365 nm. All the fluorescence spectra were measured on a Hitachi F-4600 spectrophotometer with same setting at room temperature.

4. Preparation of the nanoparticles

The nanoparticles were prepared using a well-documented reprecipitation method.

For **NID** nanoparticles: 5 microliters of 50 μM **NID** in THF solution was quickly dropped into 5 mL of deionized water under vigorous stirring at room temperature for 10 min. Afterward, the nanoparticles suspension was sonicated for another 10 min under room temperature.

For **NID-TPP** nanoparticles: 5 microliters of 50 μM **NID-TPP** in DMSO solution was quickly dropped into 5 mL of deionized water under vigorous stirring at room temperature for 10 min. Afterward, the nanoparticles suspension was sonicated for another 10 min under room temperature.

5. Cell imaging

The one-photo confocal luminescence imaging was carried out on an Olympus IX81 laser scanning confocal microscope equipped with a 40 immersion objective len. A semiconductor laser at 405 nm was served as excitation. The HeLa cells were incubated with **NID-TPP** (10 μM) for 2 h at 37°C. Then the cells were washed with PBS (phosphate buffer saline) for three times and transferred into Live Cell Imaging System (OLYMPUS, Xcellence) for confocal luminescence imaging.

The time-gated luminescence imaging setup is integrated with Olympus IX81 laser scanning confocal microscope. The luminescence signals were detected by confocal microscope system and the correlative calculation of the data was performed using professional software which was provided by PicoQuant GmbH. The excitation light of 405 nm with a frequency of 0.5 MHz from the pulse diode laser (PicoQuant, PDL 800-D) was focused onto the sample with a 40 X objective lens (NA = 0.95) for single-photon excitation.

The two-photon luminescence imaging of HeLa cells which were labeled with 10 μM **NID-TPP** were performed on a spectral confocal and multiphoton microscopes (Carl Zeiss, LSM 780 NLO) with a 40 × water objective, and a numerical aperture (NA = 1.0). The two-photon fluorescence microscopic images were obtained by exciting **NID-TPP** with a mode-locked titanium-sapphire laser source (Mai Tai HP, Spectra Physics, 80 MHz pulse frequency, 100 fs pulse width) set at the wavelength of 810 nm.

6. Cytotoxicity test

The cytotoxicity was measured using a standard methyl thiazolyl tetrazolium (MTT, Sigma Aldrich) assay in HeLa cell lines. Briefly, cells growing in log phase were seeded into 96-well cell culture plate at 1×10^4/well. **NID-TPP** was added to the wells of the treatment group at concentrations of 5, 10, 75 and 100 μM. The cells were incubated for 10 h at 37°C under 5% CO_2. The combined MTT/PBS solution was added to each well of the 96-well assay plate and incubated for an additional 4 h. After removal of the culture solution, 200 μL DMSO was added to each well, shaking for 10 min at shaking table. An enzyme-linked immunosorbent assay (ELISA) reader was used to measure the OD570 (absorbance value) of each well referenced at 490 nm. The following formula was used to calculate the viability of cell growth:

Viability (%) = (mean of absorbance value of treatment group/mean of absorbance value of control) × 100%.

7. Flow cytometry analysis

For the assay of uptake of **NID-TPP**, HeLa cells were seeded into 12-well plates (1.0×10^5/well) in 1.0 mL of completed dulbecco's modified eagle medium for 12 h. 5 μM **NID-TPP** were incubated with the cells for 30 min into the culture medium. The cells were carefully washed by PBS thrice, then harvested and resuspended with PBS, and the cellular uptake efficiency was determined on a flow cytometer (Guava, Easy-Cyte).

The supplementary figures, table and spectra

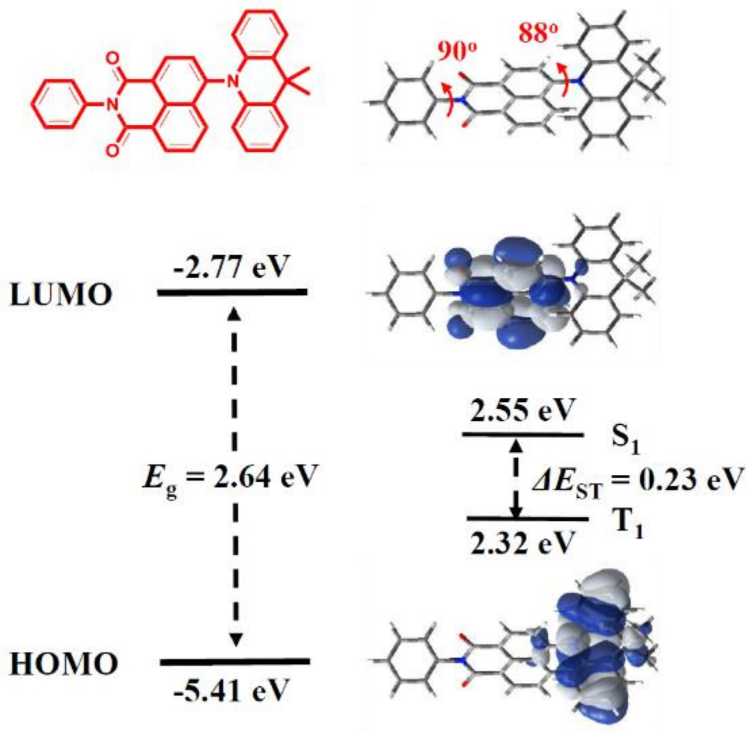

Figure S1. The ground state structure and frontier molecular orbitals of **NID** were obtained by B3LYP-D3(BJ) density functional method with basis set def2-SVP. Time-dependent DFT with a non-empirically tuned range-separated functional LC-ωPBE* with basis set def2-SVP was then performed to further analyze the excited state.

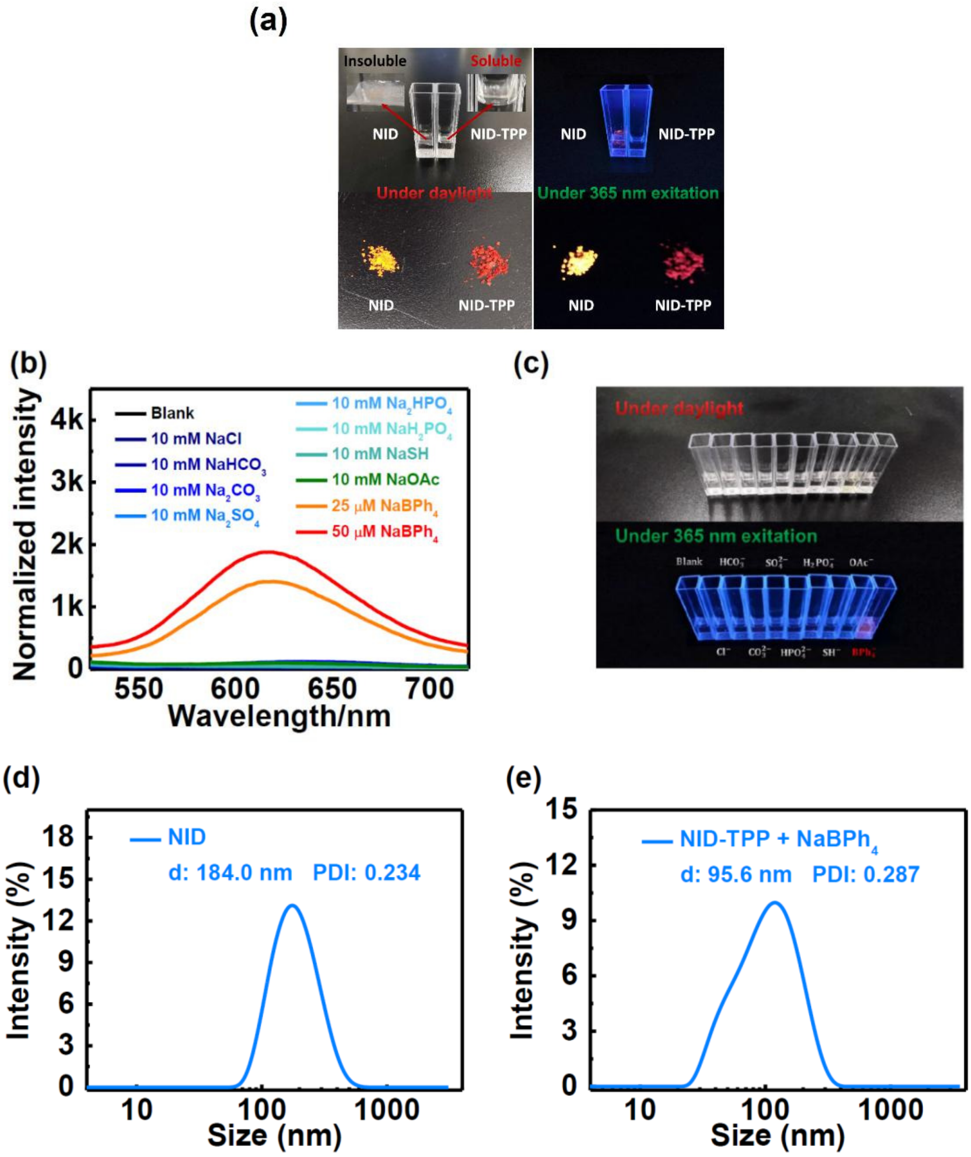

Figure S2. The upper side images are the photos of 1 mL water after the addition of 1μL of 50 μM **NID** THF solution or 1μL of 50 μM **NID-TPP** DMSO solution under daylight and UV light; the lower side images are the photos of **NID** or **NID-TPP** solid under daylight and UV light (a). The fluorescence spectra (b) and images (c) of 50 μM **NID-TPP** aqueous solution with and without the addition of different anions at room temperature (λ_{ex} = 365 nm). The DLS and PDI measurements of **NID** nanoparticles in water (d). The DLS and PDI measurements of nanoparticles of **NID-TPP** with the addition $NaBPh_4$ in water (e).

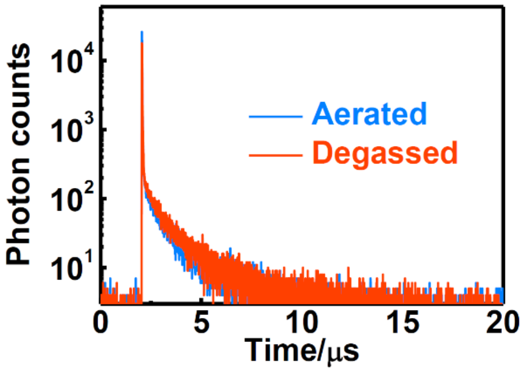

Figure S3. The transient photoluminescence decay spectra of **NID-TPP** in water with the addition of $NaBPh_4$ at room temperature under aerated (blue) and degassed (red) conditions. [**NID-TPP**] = [$NaBPh_4$] = 50 μM. λ_{ex} = 377 nm, λ_{em} = 618 nm. Under degassed condition: τ_1 = 8.9 ns (63%), τ_2 = 1.2 μs (37%), χ^2 = 1.591. Under aerated condition: τ_1 = 8.9 ns (63%), τ_2 = 1.2 μs (37%), χ^2 = 1.758.

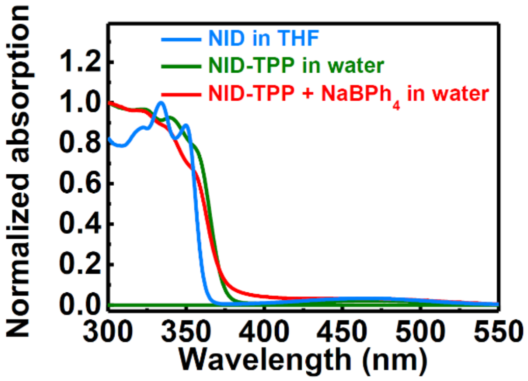

Figure S4. Normalized absorption spectra of **NID** and **NID-TPP**. [**NID**] = [**NID-TPP**] = [$NaBPh_4$] = 50 μM.

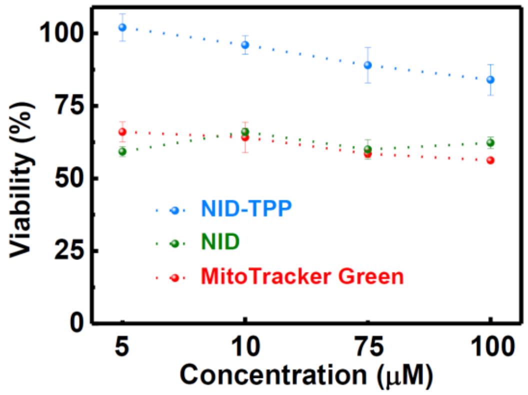

Figure S5. Cell viability values (%) assessed using an MTT proliferation test versus incubation different concentrations of **NID-TPP**, **NID** and MitoTracker Green. HeLa cells were cultured in the presence of 5-100 μM compound at 37°C for 10 h.

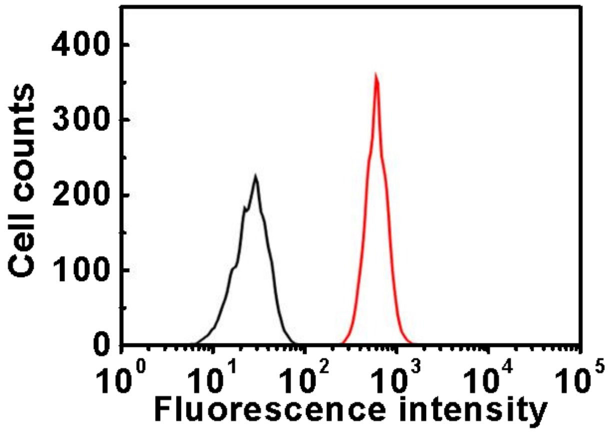

Figure S6. Flow cytometry analysis of HeLa cells incubated with (red line) or without (black line) 5 μM **NID-TPP** for 30 min at 37ºC.

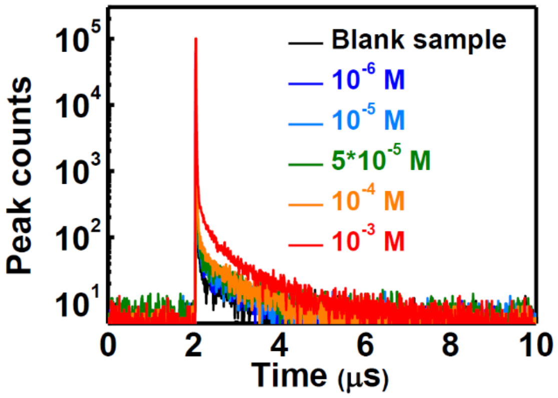

Figure S7. The transient photoluminescence decay spectra of different concentration of **NID-TPP** in water under aerated atmosphere. λ_{ex} = 377 nm, λ_{em} = 637 nm.

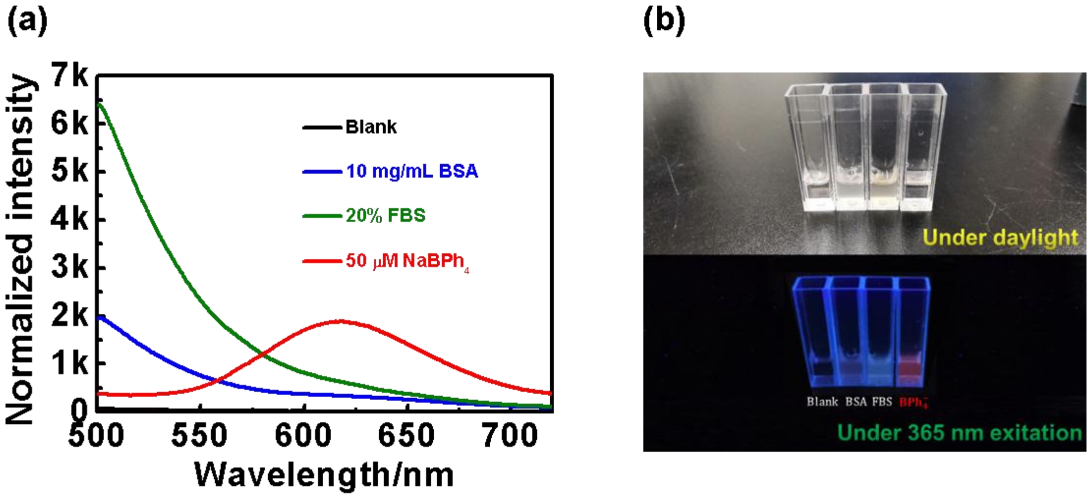

Figure S8. The emission spectra (a) and images (b) of **NID-TPP** in aqueous solution in the absence and presence of BSA (Bovine Serum Albumin), FBS (Fetal Bovine Serum) and $NaBPh_4$ at room temperature under an ultraviolet light excitation (λ_{ex} = 365 nm). [**NID-TPP**] = [$NaBPh_4$] = 50 μM, [BSA] = 10 mg/mL, 20% FBS aqueous solution.

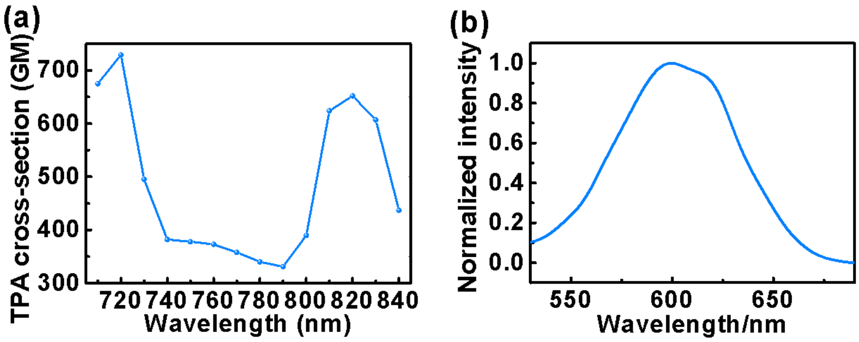

Figure S9. (a) Two-photon active cross section (Φ_δ) of 50 μM **NID-TPP** in water after the addition of 50 μM $NaBPh_4$ (λ_{ex} = 710-840 nm). (b) Two-photon emission spectra of 50 μM **NID-TPP** in water after the addition of 50 μM $NaBPh_4$ (λ_{ex} = 810 nm).

Table S1. The comparison between **NID-TPP** with the existing bench marks for TADF-based TRLI.

Compound	Small molecule or nanoparticle	Structural complexity	Specific targeting	Hydrophobic or hydrophilic	Reference
DCFMPYM	small molecule	complicated	No	Hydrophilic	4
DCFMPYM-thiol	small molecule	complicated	Yes	Hydrophilic	5
CPy	nanoparticle	simple	Yes	Hydrophobic	6
3	small molecule	complicated	No	Hydrophobic	7
BP-PTZ<br>BP-2PTZ	nanoparticle	simple	No	Hydrophobic	8
PXZT	small molecule	simple	No	Hydrophobic	9
NID-TPP	small molecule	simple	Yes	Hydrophilic	This work

Compound	AIEE	AIDFE	Additional assistance or pre-embedding	Reference
DCFMPYM	No	No	Yes	4
DCFMPYM-thiol	No	No	Yes	5
CPy	Yes	No	Yes	6
3	No	No	Yes	7
BP-PTZ<br>BP-2PTZ	Yes	No	Yes	8
ZnPXZT1	Yes	Yes	No	9
NID-TPP	Yes	Yes	No	This work

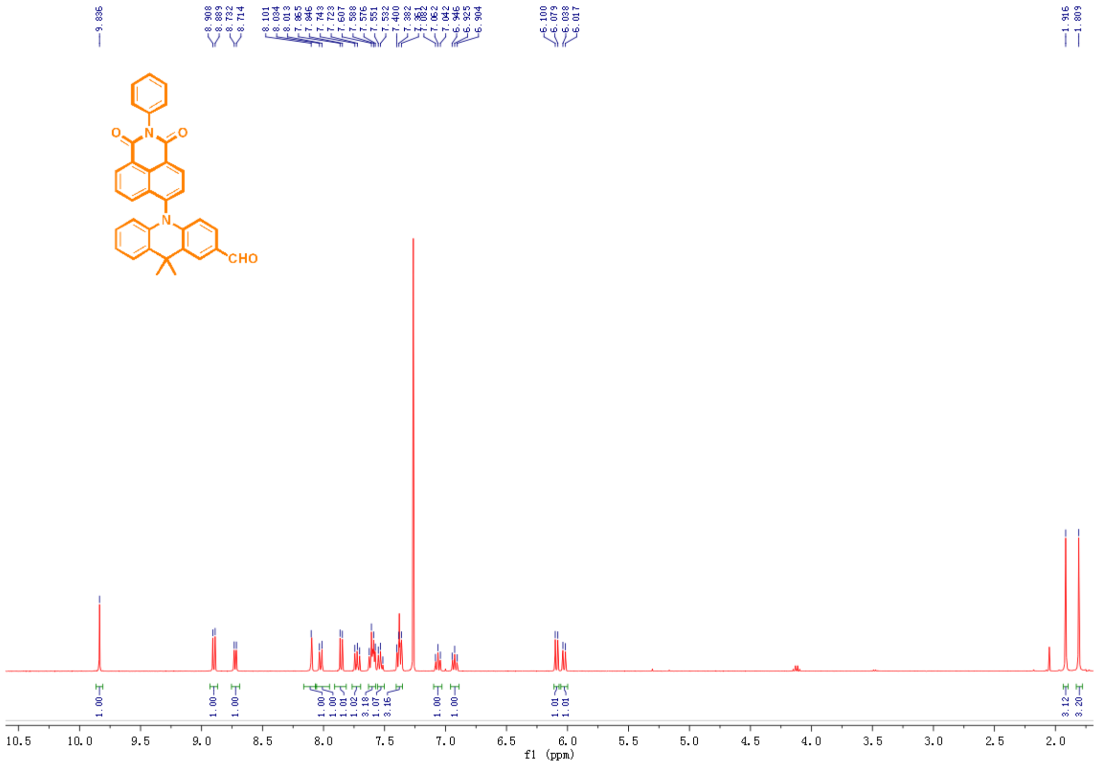

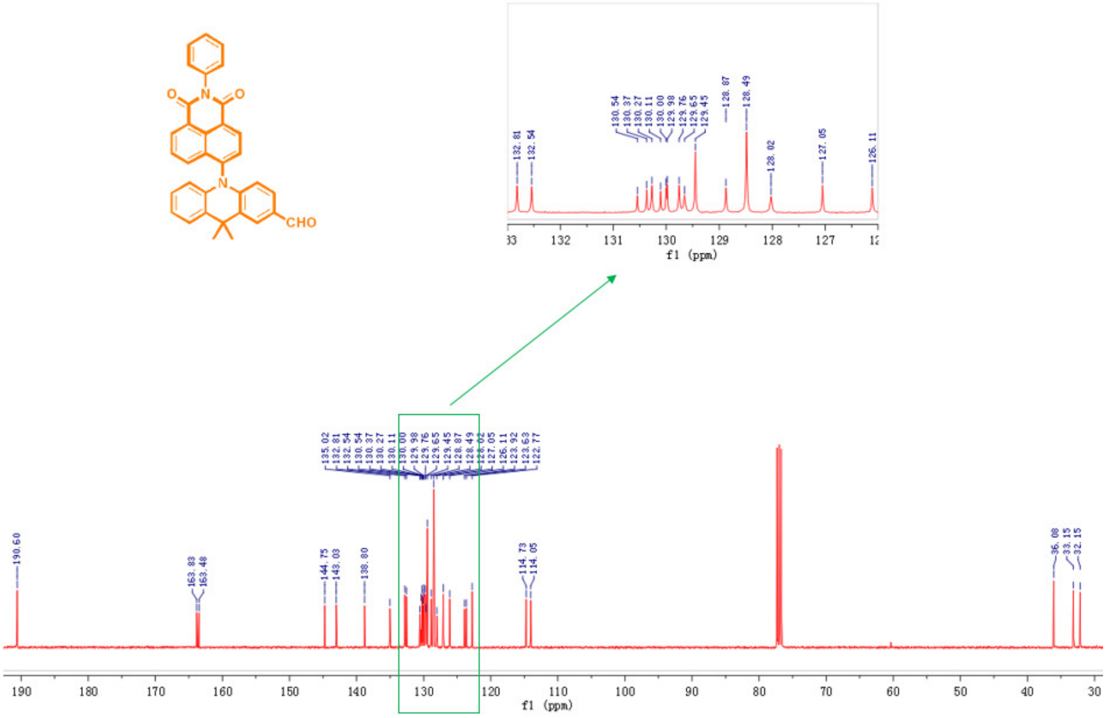

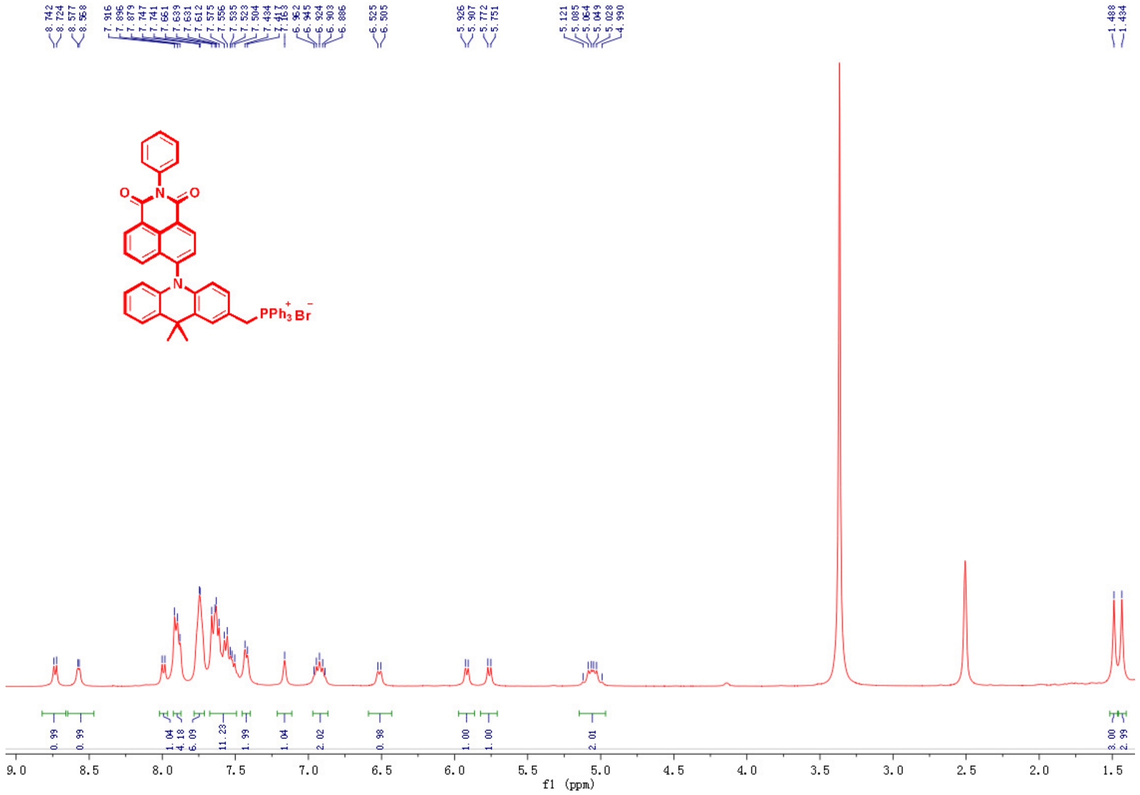
8.742 8.724 8.577 8.568 7.916 7.896 7.879 7.747 7.741 7.661 7.639 7.631 7.612 7.575 7.556 7.535 7.523 7.504 7.434 7.417 7.163 6.962 6.945 6.924 6.903 6.886 6.525 6.505 5.926 5.907 5.772 5.751 5.121 5.085 5.064 5.049 5.028 4.990 1.488 1.434
O N O
N
PPh3 Br
0.99 0.99 1.04 4.18 6.09 11.23 1.99 1.04 2.02 0.98 1.00 1.00 2.01 3.00 2.99
f1 (ppm)

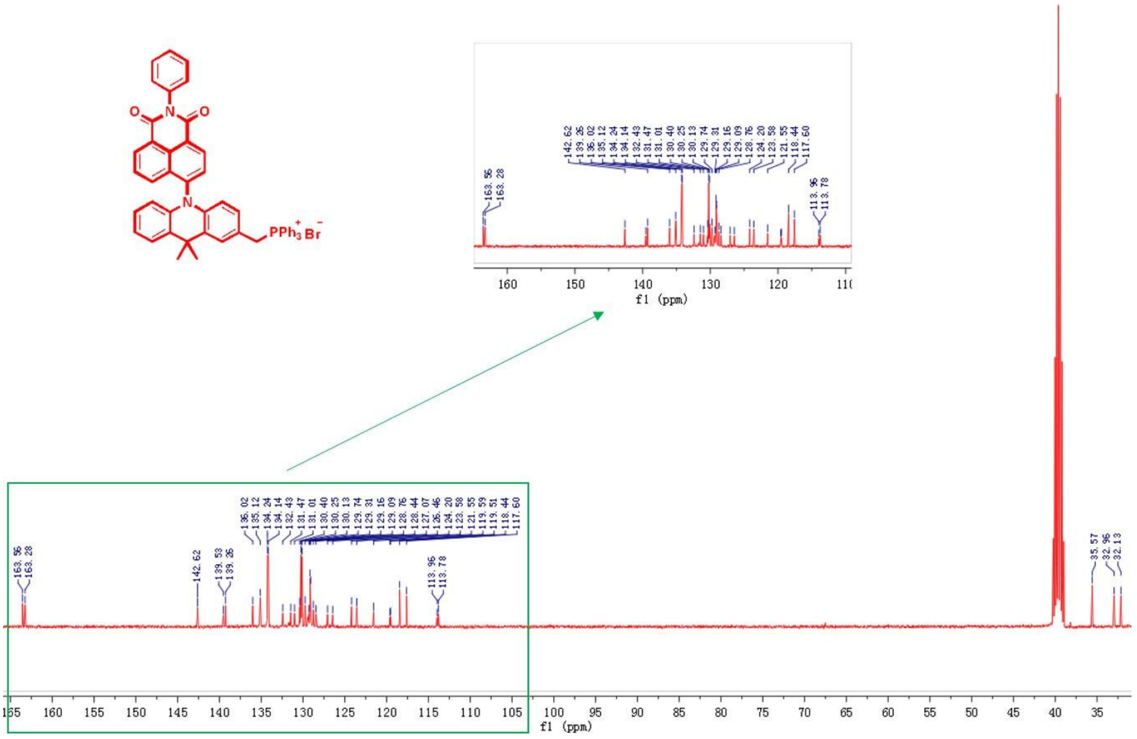
O N O
N
PPh3 Br
142.62 139.26 136.02 135.12 134.24 134.14 132.43 131.47 131.01 130.40 130.25 130.13 129.74 129.31 129.16 129.09 128.76 124.20 123.58 121.55 118.44 117.60
163.95 163.28
113.96 113.78
f1 (ppm)
136.02 135.12 134.24 134.14 132.43 131.47 131.01 130.40 130.25 130.13 129.74 129.31 129.16 129.09 128.76 128.44 127.07 126.46 124.20 123.58 121.55 119.59 119.51 118.44 117.60
163.95 163.28
142.62
139.53 139.26
113.96 113.78
35.57 32.96 32.13
f1 (ppm)

References

(1) J. F. Lee and S. L. Hsu C. Polymer **2009**, 50, 5668.

(2) J. H. Yun and J. Y. Lee, Dyes Pigments **2017**, 144, 212.

(3) W. H. Melhuish, J. Phys. Chem. **1961**, 65, 229.

(4) X. Xiong, F. Song, J. Wang, Y. Zhang, Y. Xue, L. Sun, N. Jiang, P. Gao, L. Tian and X. J. Peng, J. Am. Chem. Soc. **2014**, 136, 9590.

(5) X. Xiong, L. Zheng, J. Yan, F. Ye, Y. Qian and F. Song, RSC Adv. **2015**, 5, 53660.

(6) T. Li, D. Yang, L. Zhai, S. Wang, B. Zhao, N. Fu, L. Wang, Y. Tao and W. Huang, Adv. Sci. **2017**, 4, 1600166.

(7) Y. Wu, F. Song, W. Luo, Z. Liu, B. Song and X. Peng, ChemPhotoChem **2017**, 1, 79.

(8) S. Gan, J. Zhou, T. A. Smith, H. Su,W. Luo, Y. Hong, Z. Zhao and B. Z. Tang, Mater. Chem. Front. **2017**, 1, 2554.

(9) F. Ni, Z. Zhu, X. Tong, M. Xie, Q. Zhao, C. Zhong, Y. Zou and C. Yang, Chem. Sci. **2018**, 9, 6150.
